# A study on women’s health information needs in menopausal age

**DOI:** 10.1186/s12905-021-01582-0

**Published:** 2021-12-28

**Authors:** Sadrieh Hajesmaeel-Gohari, Elaheh Shafiei, Fatemeh Ghasemi, Kambiz Bahaadinbeigy

**Affiliations:** 1 Medical Informatics Research Center, Institute for Futures Studies in Health, Kerman University of Medical Sciences, Kerman, Iran; 2 Gastroenterology and Hepatology Research Center, Institute of Basic and Clinical Physiology Sciences, Kerman University of Medical Sciences, Kerman, Iran

**Keywords:** Menopause, Health information needs, Information sources, Challenges

## Abstract

Menopause is a natural event experienced by women in middle age. To help women manage this event, it is important to identify their health information needs. A study specific questionnaire was used to identify menopausal women’s health information needs and the resources and challenges related to finding information about menopause. A total of 301 women aged 48–55 years completed the questionnaire. Data were analysed using negative binomial regression and chi-square tests. The most frequently sought information was that related to breast cancer (*n* = 209, 69.5%), hot flushes (*n* = 200, 66.5%), cervical cancer (*n* = 194, 64.5%), non-hormonal therapies for menopausal symptoms (*n* = 192, 64%), laboratory tests (*n* = 189, 63%) and joint and muscle pain (*n* = 188, 62.5%). The main sources of information were audiovisual media (*n* = 171, 57%), obstetricians (*n* = 165, 55%), friends (*n* = 157, 52%), family (*n* = 157, 52%) and the internet (*n* = 153, 51%). The two main challenges were not knowing how to correctly access information (*n* = 115, 38%) and not being aware of reliable sources of information (*n* = 108, 36%). Therefore, it is essential for policymakers and decision-makers to provide reliable and accurate information to increase awareness and reduce anxiety of women experiencing menopause.

## Introduction

Menopause results from a cease in ovarian follicular function and marks the end of menstruation. Clinically, menopause is diagnosed after 12 months of amenorrhea [1]. Menopause occurs either naturally or is surgically or medically induced. The number of middle-aged women is growing rapidly. In 1990, the number of women aged 50 years and over was estimated at 467 million globally, and this is expected to increase to about 1200 million by 2030 [2]. Most women experience menopause between the ages of 40 and 58 years [3]. The mean age of menopause is 51 years [3], and in Iran it is 48 years [4]. Given their increased life expectancy, women are currently spending around a third of their lives in postmenopause, potentially affecting their health and quality of life [5].

The risk of non-communicable diseases such as cardiovascular disease, diabetes, chronic respiratory disease and cancer increases following menopause [6]. Studies show that non-communicable diseases affect more women than men [7] and are the main cause of death in women globally [8]. Improving women’s knowledge by providing equitable and easy access to reliable information could help reduce the rate of non-communicable diseases and improve their health [9].

Most women experience some symptoms during menopause. Vasomotor symptoms such as night sweats and hot flushes are the only symptoms specifically linked to menopause [10, 11], affecting 60–80% of menopausal women [12]. The majority of women rate these symptoms as moderate to severe [13]. Factors that can affect the severity of these symptoms include sociodemographic characteristics, lifestyle factors, psychological status and being in a dyadic relationship [14]. The provision of effective and reliable educational materials can strongly encourage menopausal women to engage in self-care and personally manage or treat their symptoms [15], improving their health and quality of life [16]. Studies show that most women have a poor understanding of menopause [17, 18]. According to a study in Iran, only about 1% of middle-aged women have satisfactory knowledge about menopause [19].

To inform women about menopause, it is first necessary to identify their information needs. Trudeau et al. [20] found that the most frequent information needs of postmenopausal women are related to menopausal symptoms, how to manage them and alternative therapies. Women seek information about managing the effects of menopause from a variety of sources, including friends, family, healthcare providers, television, radio, books, pamphlets, videos and the internet [20–22].

Studies on the information needs of menopausal women are limited, and none have previously been conducted in Iran. The purpose of this study is to investigate the information needs of menopausal women in Iran, their knowledge about menopause and interest in learning more, the sources they typically use and the challenges they face in finding information.

## Methods

### Participants

This cross-sectional study was conducted in Iran from September 2020 to March 2021. The initial sample included 258 women aged 48–55 years; however, to reduce sampling error, the sample size was increased to 301 women. Cochran’s formula (*z* = 1.96, *p* = 0.41, *q* = 0.59, *d* = 0.06) was used to calculate sample size. The *p* value was obtained from a previous study [20].

### Questionnaire design

Data were collected using a study specific questionnaire, the design of which was based on those used in similar studies [20, 22–24] and the expert opinions of two obstetricians. The questionnaire was evaluated and approved by two health informatics specialists with a background in general medicine, two health information management specialists and two obstetricians. Questionnaire reliability and internal consistency was confirmed (Cronbach’s alpha = 0.93). Fifteen participants were selected and asked to test the questionnaire. Based on the results of the pilot test, there was no need to change the content of the questionnaire.

The questionnaire included the following five sections:

- Section 1: Demographic information: age, marital status and education.
- Section 2: Three questions related to level of knowledge, level of interest in information and the frequency of searching for information about menopause, respectively. The first two questions were based on a 5-point scale ranging from *very little* to *very much*. The third question was based on a 5-point scale comprising *daily*, *weekly*, *monthly*, *rarely* and *never*.
- Section 3: Information sought by menopausal women, comprising 42 items across six categories: cancers (three items), other diseases (three items), menopausal symptoms (20 items), health care consultations (four items), diagnostic methods (four items) and treatments (eight items).
- Section 4: Sources used to acquire information about menopause (15 items).
- Section 5: Challenges in searching for information about menopause (seven items).

Sections 3–5 presented *yes* or *no* options. An open question at the end of the questionnaire invited participants to provide any additional information.

### Data collection

The questionnaire was distributed to participants either on paper or electronically. Most questionnaires were distributed to women 48–55 years of age attending the Arad Medical Centre (either as a patient or as an accompanying person). The centre offers a range of medical specialties, including gynaecology, in the city of Kerman. After explaining the purpose of the study and assuring potential participants’ of data confidentiality, informed consent was obtained. Further, because of the COVID-19 pandemic and the need for social distancing, a link to an online questionnaire was distributed via WhatsApp and Telegram. Data collection continued until the estimated sample size (*N* = 301) was met.

### Data analysis

Data were analysed by means of descriptive and analytic statistics. The Negative binomial regression model was used to compare the differences between the mean number of information items that women may need to know during menopause in every six groups and different groups of age, marital status, and educational status. Married women aged 48–51 years and without a high school diploma comprised the reference group. A chi-square test was used to examine the association between age, marital status and education and the level of knowledge, the level of interest in obtaining information, the level of need for information about menopause, the sources used to acquire information and challenges in searching for information. Data were analysed using SPSS version 22.0.

## Results

### Demographic characteristics

A total of 301 women aged 48–55 years completed the questionnaire. The mean age of participants was 51.5 ± 2.53 years. Just over half of the women were aged 48–51 years (*n* = 152, 50.5%). Most participants were married (*n* = 226, 75%) and had no academic degree (*n* = 176, 58.5%). Table 1 shows the demographic characteristics of participants.

**Table 1:** Demographic characteristics of studied women

Characteristics	Number (%)
**Age group**	
48–51 years	152 (50.5)
52–55 years	149 (49.5)
**Marital status**	
Single	13 (4.5)
Married	226 (75)
Divorced	28 (9.5)
Widow	34 (11)
**Educational status**	
Without a highschool diploma	84 (28)
Diploma	92 (30.5)
Academic	125 (41.5)

### Knowledge, interest and need levels

More than half of the participants rated their level of knowledge about menopause as ‘somewhat’ (*n* = 155, 51.5%). More than one-third of participants were ‘somewhat’ interested in obtaining information (*n* = 123, 41%). Less than half of the respondents indicated that they ‘rarely’ needed to search for information about menopause (*n* = 136, 45%) (see Table 2). The results of the chi-square test show statistically significant relationships between age group and knowledge level and between educational status and both knowledge and interest levels (*p* < 0.05) (see Table 2).

**Table 2:** The frequency of responses about the knowledge level, interest in obtaining, and need in searching information about menopause

Question	Number (%)	*P*-value		
		Age group	Marital status	Educational status
**Knowledge level**		0.014	0.063	0.000
Very little	48 (16)			
little	53 (17.5)			
Somewhat	155 (51.5)			
Much	39 (13)			
Very much	6 (2)			
**Interest in obtaining information**		0.639	0.346	0.001
Very little	14 (4.5)			
little	33 (11)			
Somewhat	123 (41)			
Much	100 (33)			
Very much	31 (10.5)			
**Frequency of needs in searching information**		0.208	0.698	0.548
Daily	10 (3.5)			
Weekly	32 (10.5)			
Monthly	94 (31.5)			
Rarely	136 (45)			
Never	29 (9.5)			

### Information needs

Table 3 shows 42 information items that women may need to know about menopause. The information most frequently needed was that related to breast cancer (*n* = 209, 69.5%), hot flushes (*n* = 200, 66.5%), cervical cancer (*n* = 194, 64.5%), non-hormonal therapies for menopausal symptoms (*n* = 192, 64%), laboratory tests (*n* = 189, 63%) and joint and muscle pain (*n* = 188, 62.5%). The information least frequently needed was related to consultations to reduce or quit smoking (*n* = 76, 25%), infertility (*n* = 84, 28%), men’s sexual problems (*n* = 89, 29.5%), women’s sexual problems (*n* = 115, 38%), hair and nail problems (*n* = 130, 43%) and consultations about exercise (*n* = 130, 43%).

**Table 3:** The frequency of “yes” responses to information items that women may need to know about menopause

Information items	Number (%)
**Women’s cancers**	
Breast cancer	209 (69.5)
Cervical cancer	194 (64.5)
Ovarian cancer	173 (57.5)
**Diseases**	
Colon cancer	163 (54)
Uterine fibroid	178 (59)
Uterine prolapse	179 (59.5)
**Menopause symptoms**	
Hot flashes	200 (66.5)
Night sweats	147 (49)
Menstrual changes	168 (56)
Sleep pattern changes	160 (53)
Depression	173 (57.5)
Anxiety	151 (50)
Alzheimer	145 (48)
Decreased concentration	153 (51)
Urinary problems	136 (45)
Skin problems	134 (44.5)
Hair and nail problems	130 (43)
Sexual problems for women	115 (38)
Sexual problems for men	89 (29.5)
Infertility	84 (28)
Blood pressure	170 (56.5)
Diabetes	141 (47)
Obesity	165 (55)
Cardiovascular problems	151 (50)
Bone problems	164 (54.5)
Joint and muscle ache	188 (62.5)
**Consultations**	
Consultation about exercise	130 (43)
Consultation about dietary	171 (57)
Consultation about reducing or quitting smoking	76 (25)
Consultation about maintaining a healthy weight	173 (57.5)
**Diagnosis methods**	
Sonography	160 (53)
Mammography	172 (57)
Bone Mineral Densitometry	187 (62)
Laboratory tests	189 (63)
**Treatments**	
A variety of treatments to reduce menopausal symptoms	187 (62)
Non-hormonal therapies to reduce menopausal symptoms	192 (64)
Hormonal therapies to reduce the symptoms of menopause	138 (46)
Complementary therapies (traditional medicine, acupuncture)	173 (57.5)
Medicinal supplements (calcium tablets, …)	184 (61)
Side effects of treatments	142 (47)
Cost of treatment	128 (42.5)
Duration of treatment	129 (43)

The mean number of selected information items in all categories (except consultations) was higher in the 52–55-year age group than in the 48–51-year age group (*p* < 0.05). The mean number of selected information items in all categories (except consultations) was significantly higher for widowed women than for married women. Moreover, compared with married women, divorced women sought more information on diseases, menopausal symptoms and treatments (*p* < 0.05). Single women required more information about treatments. Women with academic degrees required more information about all categories compared with those without (see Table 4).

**Table 4:** The Negative binominal regression model to compare the differences between the mean numbers of information needed items

	Mean ± S.D	Regression coefficient* (95% confidence interval)	*P*-value
**Women’s cancers**			
**Age group**			
48–51	2.27 ± 1.63	Reference	Reference
52–55	1.85 ± 1.61	1.23 (1.06,1.41)	0.004
**Marital status**			
Married	1.77 ± 1.20	Reference	Reference
Single	2.17 ± 1.08	1.22 (0.93,1.62)	0.147
Divorced	2.04 ± 1.25	1.15 (0.91,1.46)	0.226
Widow	2.25 ± 1.35	1.27 (1.01,1.60)	0.039
**Educational status**			
Without a highschool diploma	1.80 ± 1.50	Reference	Reference
Diploma	2.03 ± 1.61	1.12 (0.91,1.39)	0.262
Academic	2.35 ± 1.53	1.30 (1.07,1.59)	0.009
**Diseases**			
**Age group**			
48–51	1.75 ± 1.49	Reference	Reference
52–55	2.02 ± 1.51	1.51 (0.99,1.33)	0.058
**Marital status**			
Married	1.57 ± 1.12	Reference	Reference
Single	1.70 ± 1.04	1.08 (0.77,1.51)	0.650
Divorced	2.28 ± 0.77	1.45 (1.25,1.69)	0.000
Widow	2.06 ± 1.30	1.31 (1.03,1.67)	0.024
**Educational status**			
Without a highschool diploma	1.72 ± 1.36	Reference	Reference
Diploma	1.84 ± 1.44	1.07 (0.87,1.31)	0.514
Academic	2.10 ± 1.53	1.22 (0.99,1.49)	0.052
**Menopause symptoms**			
**Age group**			
48–51	9.43 ± 8.75	Reference	Reference
52–55	11.96 ± 9.39	1.26 (1.09,1.47)	0.002
**Marital status**			
Married	8.76 ± 6.31	Reference	Reference
Single	9.13 ± 6.22	1.04 (0.71,1.52)	0.831
Divorced	12.50 ± 5.76	1.42 (1.17,1.72)	0.000
Widow	12.74 ± 7.28	1.45 (1.17,1.80)	0.001
**Educational status**			
Without a highschool diploma	10.24 ± 7.78	Reference	Reference
Diploma	9.52 ± 8.43	0.93 (0.75,1.14)	0.486
Academic	12.31 ± 9.50	1.20 (0.99,1.45)	0.057
**Consultations**			
**Age group**			
48–51	1.77 ± 1.80	Reference	Reference
52–5	1.98 ± 1.86	1.11 (0.93,1.32)	0.218
**Marital status**			
Married	1.64 ± 1.32	Reference	Reference
Single	1.69 ± 0.91	1.03 (0.76,1.39)	0.849
Divorced	2.08 ± 1.56	1.27 (0.95,1.69)	0.101
Widow	2.14 ± 1.88	1.30 (0.94,1.80)	0.103
**Education**			
Without a highschool diploma	1.58 ± 1.56	Reference	Reference
Diploma	1.69 ± 1.66	1.06 (0.82,1.38)	0.618
Academic	2.45 ± 1.99	1.55 (1.23,1.94)	0.000
**Diagnosis methods**			
**Age group**			
48–51	2.15 ± 1.92	Reference	Reference
52–55	2.79 ± 2.12	1.29 (1.11,1.49)	0.001
**Marital status**			
Married	2.12 ± 1.44	Reference	Reference
Single	2.29 ± 1.36	1.08 (0.77,1.50)	0.646
Divorced	2.78 ± 1.30	1.31 (1.08,1.58)	0.004
Widow	2.67 ± 1.60	1.26 (1.01,1.57)	0.036
**Education**			
Without a highschool diploma	2.31 ± 1.70	Reference	Reference
Diploma	2.06 ± 1.84	0.89 (0.72,1.09)	0.278
Academic	3.10 ± 2.00	1.34 (1.14,1.57)	0.000
**Treatments**			
**Age group**			
48–51	4.32 ± 3.79	Reference	Reference
52–55	5.28 ± 3.87	1.22 (1.03,1.44)	0.016
**Marital status**			
Married	3.75 ± 2.88	Reference	Reference
Single	5.15 ± 2.31	1.37 (1.06,1.78)	0.016
Divorced	5.24 ± 2.85	1.40 (1.12,1.74)	0.003
Widow	5.15 ± 3.39	1.37 (1.07,1.76)	0.011
**Education**			
Without a highschool diploma	4.41 ± 3.62	Reference	Reference
Diploma	4.38 ± 3.44	0.99 (0.79,1.24)	0.958
Academic	5.65 ± 4.22	1.28 (1.04,1.57)	0.019

*Exponentiation of the B coefficient (EXP (B))

### Information sources

Table 5 shows that 15 sources were used to obtain information. The most frequently used sources were audiovisual media (*n* = 171, 57%), obstetricians (*n* = 165, 55%), friends (*n* = 157, 52%), family (*n* = 157, 52%) and the internet (*n* = 153, 51%). The least frequently used sources were religious groups (*n* = 22, 7.5%), workshops (*n* = 28, 9%), pharmacists (*n* = 29, 9.5%), nurses (*n* = 49, 16.5%) and educational videos (*n* = 60, 20%).

**Table 5:** The frequency of sources used to acquire information about menopause

Source	Number (%)	*p*-value		
		Age group	Marital status	Educational status
Written sources (books, brochures, magazines, newspapers, articles)	119 (39.5)	0.056	0.593	0.000
Audio-visual media (TV, radio)	171 (57)	0.000	0.025	0.040
Educational videos	60 (20)	0.069	0.365	0.045
Telephone consultation	52 (17.5)	0.005	0.516	0.067
Internet (websites and social networks)	153 (51)	0.602	0.044	0.000
Family	157 (52)	0.001	0.050	0.434
Friends	157 (52)	0.056	0.108	0.643
Obstetricians	165 (55)	0.031	0.432	0.114
General Practitioners	78 (26)	0.248	0.756	0.209
Midwives	79 (26.5)	0.001	0.449	0.342
Pharmacists	29 (9.5)	0.027	0.399	0.155
Nurses	49 (16.5)	0.016	0.454	0.143
Specialists in traditional medicine or herbalists	102 (34)	0.002	0.365	0.908
Workshops	28 (9)	0.100	0.633	0.206
Religious groups	22 (7.5)	0.168	0.097	0.356

There were statistically significant relationships between age group and the use of written sources, audiovisual media, telephone consultations, family, friends, obstetricians, midwives, pharmacists, nurses and traditional medicine specialists or herbalists (*p* < 0.05). Significant relationships were also found between marital status and seeking information from audiovisual media, the internet and family members and between educational status and seeking information from written sources, audiovisual media, educational videos, the internet and groups (*p* < 0.05).

### Challenges in finding information

Table 6 shows seven challenges women face when searching for information about menopause. The most frequent challenges faced by participants were not knowing how to correctly access information (*n* = 115, 38%) and not being aware of reliable sources of information (*n* = 108, 36%). The least frequent challenges were menopausal symptoms (*n* = 67, 22.5%) and limited access to information (*n* = 82, 27%).

**Table 6:** The frequency of challenges in searching information about menopause

Challenge	Number (%)	*P*-value		
		Age group	Marital status	Educational status
Information from different sources is contradictory	106 (35)	0.040	0.114	0.329
I do not have enough time to search information	106 (35)	0.187	0.788	0.916
I do not find enough information	103 (34)	0.806	0.124	0.040
I do not know the sources of information	108 (36)	0.897	0.028	0.131
I do not know how to access information correctly	115 (38)	0.466	0.481	0.359
I have limited access to information (library, internet, etc.)	82 (27)	0.055	0.077	0.022
Menopausal symptoms prevent the search for information (Decreased memory or concentration, lack of energy, mood swings, etc.)	67 (22.5)	0.548	0.909	0.858

A statistically significant relationship was found between age group and the following two challenges: contradictions between different sources and limited access to information (*p* < 0.05). Significant relationships were also found between marital status and being aware of sources of information and between educational status and not finding enough information and limited access to information (*p* < 0.05).

## Discussion

This study was conducted to identify the health information needs of menopausal women as well as the sources used to seek information and the challenges faced while searching for information.

Most participants sought information about breast cancer, hot flushes, cervical cancer, non-hormonal therapies, laboratory tests and joint and muscle pain. This finding is similar to that of a study conducted on midlife women in Singapore [23], which showed that gynaecological cancers, joint and muscle pain, bone health and breast cancer testing were the most commonly selected topics. Breast cancer is the most common cancer in women worldwide, including in Iran. Hormonal replacement therapy following menopause is a risk factor for breast cancer [25]. While hormonal therapies are effective in the treatment of menopausal vasomotor symptoms, many women prefer non-hormonal treatments because of the side effects of hormonal therapies and their limited use in some cancers [26]. This indicates the importance of providing information about breast cancer and non-hormonal menopausal treatments.

Cervical cancer was another issue considered by participants in this study. In Iran, cervical cancer mainly occurs in postmenopausal women aged 55–65 years. The incidence of cervical cancer in Iran is 2.5 per 100,000 women, the average mortality rate is 1.04 per 100,000, and the mortality-to-incidence ratio is 42% [27]. These statistics and the findings of the present study reveal the importance of providing sufficient information about cervical cancer and its risk factors to reduce its prevalence.

Participants also frequently sought information about hot flushes and joint and muscle pain, two of the most common symptoms experienced by menopausal Iranian women [28, 29], which provides a possible explanation for our results.

Information about treatments to reduce or quit smoking, infertility and men and women’s sexual problems was sought less frequently. The prevalence of smoking in Iran is moderate compared with other Asian countries—only 3% of Iranian women smoke cigarettes [30], which may explain why few women search for information about reducing or quitting smoking. Seeking information about sexual issues may be less common because of the shame or taboo associated with these issues in the Iranian sociocultural context. Shame is frequently a barrier to seeking health information for women [31]. However, sexual problems increase with age, a decrease in oestrogen levels and menopause and can affect life satisfaction [32, 33].

The most frequently used information sources were audiovisual media, obstetricians, friends, family and the internet. In contrast, a study conducted on middle-aged Brazilian women found that the most frequently used sources of information were friends, family, physicians and audiovisual media, while the least frequently used source was the internet [21]. However, this difference is most likely related to the data being gathered in 2012 and 2013, meaning that public access to the internet was limited. Moreover, there were few discussions about menopause in the Brazilian media, which was used much less as a source compared with friends, family and physicians [21].

The most frequent challenges in finding information were related to not knowing how to access information and not being aware of reliable sources of information. This shows the need for healthcare providers to educate women about menopause. Educational programs could increase women’s knowledge about this critical topic [17, 20]. A previous study on women’s information needs about complementary and alternative treatments for menopause symptoms showed contrasting results [24]. In this study, the greatest challenges were information gaps, contradictory information and limited time to search for information. These differences may be related to the fact that the study investigated challenges in searching for information about a particular treatment for menopausal symptoms, while our study investigated challenges in searching for information about all aspects of menopause.

More than half of the participants in our study rated their level of knowledge about menopause as ‘somewhat’. This result is consistent with a previous study that found that most women had moderate knowledge about menopause [19]. We also found that women with academic degrees had more knowledge and interest in obtaining information about menopause. A study on Jordanian women’s knowledge about menopause showed the same result [34]. It is likely that women with an academic degree have more knowledge and interest in finding information about menopause because they are more capable of searching for reliable information.

To the best of our knowledge, this is the first study on the information needs of menopausal women in Iran. However, it has some limitations. Because of the COVID-19 pandemic, participation in the questionnaire was less than expected, meaning that we had to create an online version. This may have influenced the findings, especially the finding related to the internet as one of the most frequently used information sources.

## Conclusion

Menopause is a natural stage in the life of middle-aged women and can have a significant impact on quality of life. Identifying the information needs of menopausal women can help in the design of practical training programs, raise levels of awareness and improve quality of life. The findings of this study show that women require various types of information, particularly information about cancers, the clinical signs of menopause and non-hormonal therapies for menopausal symptoms. Therefore, it is important that health policymakers and decision-makers provide reliable and accurate information about these aspects to improve women’s understanding of menopause and help ensure peace of mind.

## Data Availability

The datasets used and/or analysed during the current study available from the corresponding author on reasonable request.
